# Imaging Features of Calciphylaxis on Conventional Radiography and Computerized Tomography

**DOI:** 10.5334/jbsr.3784

**Published:** 2025-02-08

**Authors:** David Willain, Thomas Kirchgesner, Lokmane Taihi

**Affiliations:** 1 Cliniques universitaires Saint‑Luc, Belgium; 2 Radiology department, Cliniques universitaires Saint‑Luc, Belgium

**Keywords:** Calciphylaxis, vascular calcifications, skin ulcers, CT, mammography, conventional

## Abstract

We report a case of a dialysis patient with painful leg ulcers and a clinical suspicion of calciphylaxis. Conventional radiography revealed extensive hypodermic vascular calcifications, characteristic of the disease. A computed tomography (CT) was performed and objectified the progression of the disease within a few years. This case underscores the value of conventional radiography in cases of suspicion of calciphylaxis and the added value of CT.

## Case History

A 70‑year‑old female dialysis patient presented with painful ulcers on the lower limbs. Clinical examination revealed indurated plaques accompanied by livedo of the limbs. Given her medical history and the clinical presentation, calciphylaxis was suspected.

**Figure 1:**
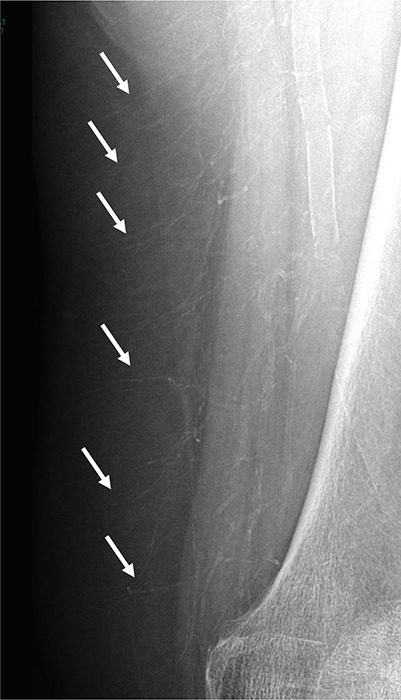
X‑ray revealing extensive vascular calcification of femoral arteries, with extensive involvement of smaller arterial branches, tracing the microvascular network.

X‑rays revealed extensive vascular calcification of femoral arteries, with extensive involvement of smaller arterial branches, tracing the microvascular network and affecting not only intramuscular but also hypodermic arteries oriented perpendicular to the skin’s surface, creating an image reminiscent of a pseudo‑arteriogram (Figure 1).

**Figure 2:**
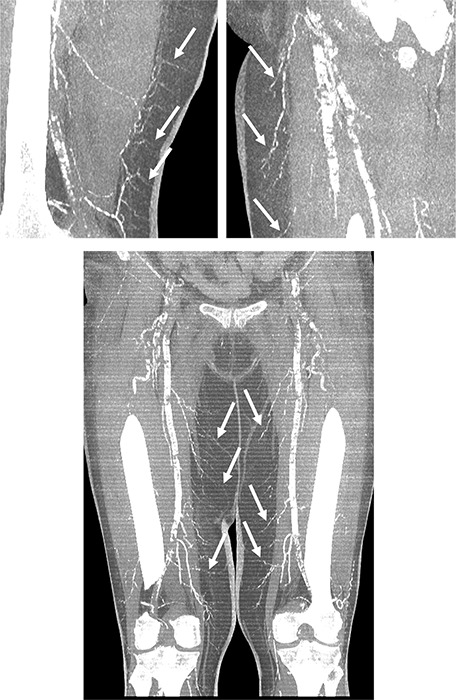
MIP reconstructions showing detailed visualization of the hypodermic arterial calcifications.

**Figure 3:**
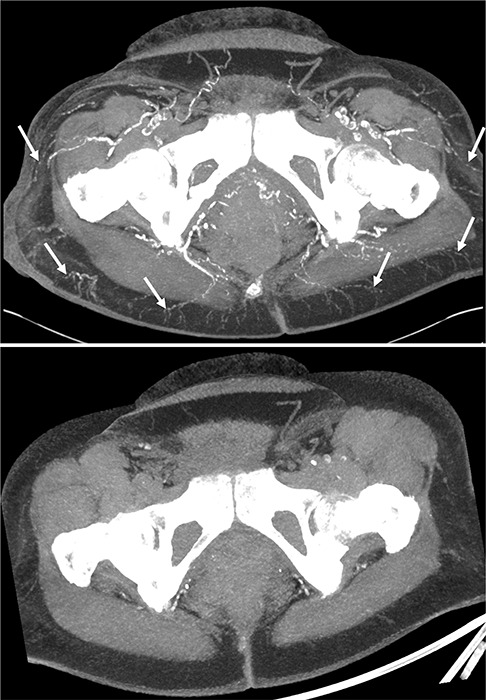
MIP reconstructions showing the rapid evolution of the arterial calcifications within two years.

A computed tomography (CT) was also performed and showed detailed visualization of the hypodermic arterial calcifications. Maximum intensity projection (MIP) images were particularly valuable in delineating the extent and distribution of calcification, showing continuous calcified segments of the distal arteries in the subcutaneous tissue. This provided a comprehensive map of the affected vascular territories, solidifying the diagnosis of calciphylaxis (Figure 2). The appearance of these calcifications, compared with an examination conducted 2 years earlier (Figure 3) was unusual for chronic calcifying diseases.

## Commentary

Calciphylaxis is a rare condition affecting primarily patients with chronic kidney disease, especially those on dialysis. It involves calcification of small to medium‑sized blood vessels in the skin, leading to compromised blood flow, tissue ischemia, and necrosis. It often manifests as painful ulcers, which begin as areas of erythema and induration with livedo, progressing eventually to necrotic lesions. Early and accurate diagnosis is essential, with radiologists playing a key role in identifying the characteristic vascular calcifications [1].

While skin biopsy has traditionally been the gold standard for diagnosing calciphylaxis due to its ability to provide definitive histological evidence, it has notable limitations. The procedure is time‑consuming and carries risks such as infection and delayed wound healing [1]. Consequently, in cases of clinical doubt, imaging techniques can be utilized for corroborating the diagnosis. In this regard, a thorough assessment of vascular calcifications can be achieved using conventional radiography and CT scans. Conventional radiographs are the first‑line imaging modality, providing effective visualization of calcifications. However, CT scans with MIP images offer superior detail, helping delineate the extent of vascular involvement, which is important for prognosis and treatment planning [1]. Mammography, while offering high resolution and contrast for detecting calcifications, can pose technical challenges when applied to the limbs. In conclusion, conventional radiography and CT may contribute to the early detection of calciphylaxis, enabling prompt diagnosis while minimizing the need for invasive procedures.
